# Historical Sketch of the Progress of Medicine in the Year 1806

**Published:** 1807-07

**Authors:** 


### Medical and Phyfical Journal.

VOL. XVIII.] JULY, 1807. [NO. 101.

*Printed for R. PHILLIPS, by W. Thorne, Red Lion Court, Fleet Street, London*

### HISTORICAL SKETCH OF THE PROGRESS OF MEDICINE IN THE YEAR 1806; BY MR. ROYSTON.

( To be continued Annually. )

AMONG the inventions that have arisen out of the intellectual powers of man, the rational exercise of the medical art is, of all others, the most connected with the welfare of society. Of this, England affords many striking examples. Down to the period of the last appearance of pestis in this country, but few years ever elapsed without some contagious disease spreading devastation and death; scarcely was there a reign in which the historian had not to record some wide-wasting pestilence, which barely left alive a number sufficient to bury the dead. But since medical science has taught the means of prevention, this dreadful havoc of the human race seldom occurs.

In the treatment of diseases of minor consequence, the discriminating theories, and the rational simplicity of modern practice, have administered to the security and comfort of mankind, in a degree always proportioned to the intelligence with which they have been employed. Reflection on these facts renders it extremely difficult to account for the comparative neglect the profession and the professors of medicine have experienced. In an age when the progression of general science hath occupied the attention of states and empires; when the arts of design have their Academies, and natural philosophy its Institutions; when music is cultivated with successful enthusiasm, and rewarded with regal munificence; when philosophical legerdemain excites applause, and the quackeries of Perkins extort wealth, it becomes a question of the first importance, why the Science of Medicine has not had a proportionate share of public approbation, and of princely reward? Is it, that mankind, ever seeking for the new and surprising, give with profuse and unsparing hand to the dextrous buffoon, who tickles the fancy, and neglects the patient investigator of the laws of nature, because he has not this amusing talent, even when his labors are exerted to sooth the pains of life; to give that health, without which the tricks of the juggler have no interest, the grimaces of the buffoon no powers of exhiliration, and the dulcet note of music no charm.

Neglect cannot, however, paralyse the ardor for philosophical investigation. If the *genuine* physician is days, and weeks, and months, laboring in his profession for a remuneration, which half an hour of time, and the warbling a favorite air, brings to a public singer; still he pursues, unwearied and uncomplaining, the duties of his avocation; solicitous to benefit mankind by his studies, to explain the extent and powers of his art, and to unfold the mysterious laws of Nature. Regardless of popular applause, and invulnerable behind the shield of rectitude to popular censure, his primary object is to disclose his discoveries, to illustrate his modes of practice, and to give publicity to the steps he has pursued in his efforts to improve his art: while the affectation of mystery, the concealed nostrum, the pretended arcanum, are the strong holds of empiricism. A desire to promulgate knowledge is a prominent trait in the character of the present age; and if the liberal motives that impel the members of a learned profession, to explain to the world the principles and the practice of their art, deserve praise; the numerous streams of professional learning, so various in extent, interest, and talent, to which this spirit of discovery has given birth, have rendered an annual history of their progress a desideratum in medical literature.

With the impression of the expediency of a periodical Epitome of the progress of Medical Discovery, and the utility of a Retrospect of Medical Literature, the following Sketch is submitted, as a specimen of an annual detail, which is intended to comprehend the publications on medical subjects, and the discoveries of the Faculty in every part of the world.

In tracing the history of the progress of a science within a stated period, the most obvious and natural course will be, to inquire what has been done to meliorate its condition generally, as one of the aggregate parts of Society; and from thence descend to particular improvements, and individual efforts. In arranging the materials of this history, patriotism will find an apology for giving the first place to England; the second will be occupied by by France, her rival in arts and arms; Spain, Portugal, Italy, Germany, and the north of Europe will follow; and the detail will close with Hindostan and America.

In the year 1806, an attempt has been made to increase the respectability of the science of medicine throughout the British empire, by reforming its abuses, correcting its irregularities, and by establishing proper tests for ascertaining the qualifications of those who apply to be admitted into its privileges. In short, to effect in it a radical Reform.

During a long series of years, a description of persons, totally uneducated, and ignorant in the extreme, have been permitted to practise the art of medicine, to the incalculable injury of mankind, until they have increased, in every district where the inquiry has been made, to a number that almost staggers belief, and which should appal those who are to be the victims. From the time of Francis Anthony, of *aurum potabile* memory, to the present hour, a history of nostrum-mongers, proprietors of patent medicines, urine-casters, astrologers, fortune-tellers, who have combined with their privilege of looking into the book of fate, the practice of medicine, with a long muster-roll of quack-salvers, &c. would make an amusing, an interesting, and a useful volume. So long since as the year 1612, Dr. John Cotta, of Northampton, published a quarto volume, with the title of a "Short Discovery of the unobserved Dangers of several Sorts of ignorant and inconsiderate Practisers of Physic in England." This is an elaborate history of the arts of Quackery down to the period of its publication. Since then, notwithstanding the general diffusion of knowledge, materials for a continuation of that work have become very abundant. This extension of Charlatanism, so remarkable in England,* may be explained, perhaps, from the facility with which intruders get into the profession, from the revenue produced by the sale of nostrums, and from the want of a precise defining of the powers of those corporate institutions, in whom the regulation of the profession is vested.

Under these existing circumstances, Dr. Edward Harrison, of Horncastle in Lincolnshire, in a circular letter,

* Dr. Adams will not allow (Med. and Phys. Journal, No. 86, p. 335) the pre-eminence of England in Empiricism. The solitary instance of quackery, in la Revue ou Decade Philosophique, by Alphonse Leroy, which professes to cure epilepsy by a green stone from the river Oroonoco, makes but a diminutive figure, when opposed to *our* hosts of specifics, and *our* miriads of panaceas.

called for the concurrence and assistance of the profession at large, in furtherance of a scheme of medical reform, which he explicitly detailed in his Remarks.* The immediate result of this, was meetings at the house of the President of the Royal Society.† The College of Physicians was applied to for its assistance in the plan suggested by Dr. Harrison; and the concurrence of the minister was expected, if not promised. The resolutions of the College of Physicians ‡ in Ireland, and the approval of various Societies, were transmitted to Dr. Harrison; but the project, at least, became stationary, either from the intellects of mankind being absorbed in the great political events passing on the stage of the world, from the coldness with which the incorporated bodies received it,|| or from the London College of Physicians having entered themselves on plans of reform, or having determined, under their present active president, to enforce the existing statutes. If the College of Physicians has come to a resolution of energetically exercising its powers, which have lately appeared to have an extent far beyond their formerly supposed limits,§ he must be a sturdy partizan who would insist that the great work of reformation is more properly placed in the hands of Doctor Harrison, and the Lincolnshire Society, than under the cognizance of that body. The faculty of Physic, however, is under some obligation to Dr. Harrison, for exciting attention to a subject,

* Remarks on the Ineffective State of the Practice of Physic in Great Britain, with Proposals for its future Regulation and Improvement; and the Resolutions of the Members of the Benevolent Society of Lincolnshire, 8vo. Lond. 1806.

† At a meeting of the Faculty, held at Sir Joseph Banks's, Aug. 9, 1806, various resolutions were entered into, a plan was formed, and a committee of correspondence appointed, (Edin. Med. Journ. ii, 489). And at a meeting of this committee, at Dr. Garthshore's, Aug. 25, 1806, a letter was addressed to the College of Physicians, requesting a conference on this important subject. Ibid. 490. Med. and Phys. Journ. Oct. 1806.

‡ Edin. Med. Journ. April, 1807, 250.

|| The Royal College of Surgeons of Edinb. in a letter to Dr. Harrison, asserts its conviction, that the powers and privileges with which the different Universities, Colleges, and Corporations of Physic and Surgery are already invested, are sufficient, if duly exercised, for the correction of the greater part of the existing abuses. Med. Journ. Feb. 1807, p. 195.

§ The Royal College of Physicians of London, having ascertained, by an examination of their archives, that they have the power to regulate the practice of physic throughout the kingdom; have manifested their intention of employing those powers by advertisements repeatedly published in the London News-papers. Edin. Med. and Surg. Journ. ii. 487.

ject, at once connected with the respectability of the practice of medicine and with the interests of society.

In the different departments of the profession, the industry of the faculty of Great Britain, has this year produced an accession to scientific knowledge, highly creditable to its application and talents. In anatomy and physiology, Mr. Saunders's work on the Ear * stands foremost. This splend'd folio, contains a very lucid description of the structure, functions, and diseases of the auditory organ; illustrated by well conceived, and finely executed plates. It begins with a description of the auricle and meatus externus; this is followed by an account of the middle part of the ear, consisting of the tympanum and the machinery contained within it. A description of the internal part succeeds, and which is to be considered as the immediate organ of hearing, because it contains the expansion of the auditory nerve. The anatomy is followed by a description of the diseases of the ear, which is rendered particularly interesting, by the few attempts that have hitherto been made to investigate the morbid changes to which this organ is liable. The causes which have occasioned the morbid anatomy of the ear to be neglected, are here forcibly pointed out; and though the difficulties of elucidating this subject are admitted, yet Mr. Saunders has demonstrated that they are not insuperable. The diseases of the meatus externus are first noticed. These are an inflammation, sometimes going on to suppuration, and followed by an exfoliation of the meatus externus of the os temporis, or of the external lamina of the mastoid process. An herpetic eruption on the meatus externus and auricle. Excrescences arising out of the lining of the meatus externus, resembling syphilitic warts, and appearing to be produced by irritation. A septum obstructing the passage of the meatus externus. And an inspissation of the cerumen. The diseases of the tympanum are next treated. The principal of these is a puriform discharge from the tympanum. This discharge is ichorous, sometimes tinged with blood, and imparts a yellow colour to a silver instrument. There is a loss of hearing proportionate to the injury which the machinery of the tympanum has sustained. The author is extremely full and explicit on

* The Anatomy of the Human Ear, illustrated by a series of engravings of the natural size, with a Treatise on the Diseases of that Organ, the Causes of Deafness, and their proper Treatment. Folio, Lond. 1806, pp. 60. plates 4.

on this disease, which is the most serious of those that attack the organ of hearing. A destruction of essential parts of the organ, ending in irremediable deafness, is among its common consequences. We have lately seen, in the case of a gentleman 60 years of age, this disease extended to the internal part of the cranium.—Inflammation, suppuration, and death ensued. On opening the head, a considerable quantity of pus was found lying between the dura and pia mater, under the parietal bone of the same side; and the connection with the disease of the ear, was clearly traced by the ingenious anatomist who performed the dissection. Mr. Saunders divides this disease into three stages. The first of these, is a simple puriform discharge; the second is a puriform discharge, with fungus and polypi; and the third is complicated with a caries of the tympanum. In each of these he distinctly details the symptoms and mode of cure. We cannot too earnestly recommend this part of the work, both from the serious nature of the disease, and from the erroneous opinions that prevail with respect to its treatment. Obstructions of the Eustachian tube are next investigated, and the work concludes with the diseases of the internal part of the ear.—The nature of the deafness which arises from disease of the internal part of the ear, is, at present, completely obscure, from our ignorance of the morbid changes which are the immediate cause of the defect. Mr. Saunders is of opinion, that this class of disease originates in a want of sensibility in the nerve, some alteration in the structure of the membranes on which the nerve is expanded, or some change in the properties of that fluid which is contained in the membranes, and which is the immediate agent in impressing the sentient extremities of the nerve. There is, however, a species of total deafness, which exists without any apparent defect in the organization of any part of the ear.

The whole class of the diseases to which the internal part of the ear is subject, may be denominated nervous; a generic term signifying every disease, the seat of which is in the nerve, or parts containing the nerve.

The plates, in number 4, give a view of the meatus externus, membrani tympani, and Eustachian tube; a section of the cranium and face, to shew at one view the relative position of the above parts; an interior view of the membrani tympani, and Eustachian tube, with a dissection of the os temporis, to shew the chain of bones between the membrana tympani and vestibule, precisely in their their proper situation; the membrana tympani, the malleus, and the tensor membrani tympani attached to it; the fœtal os temporis, the different positions of the individual bones which form the connexion between the membrana tympani and the membrane of the vestibule; the anterior portion of the mastoid process and tympanum, divided by a vertical section, to exhibit the mastoid cells, the internal surface of the mem. tympani, and the portio dura of the auditory nerve; the interior portion of the mastoid process, the interior part of the tympanum, and the Eustachian tube; the interior superficies of the tympanum, dissected to show the stapedeus muscle; the skeleton of the interior superficies of the tympanum, that the fenestra ovata and rotunda may be seen; the internal parts of the ear, labyrinth, cochlea, &c.

If Mr. Saunders, in applying to a subject on which little has hitherto been done, has had many difficulties to encounter, which the industry and study of others might have removed; he has acquired additional credit by exploring an unknown region, by elucidating an obscure subject, and by establishing points of practice, before, loosely, or erroneously understood.

Mr. Hunt's "Anatomical Reflections"* is a disquisition that may not, perhaps, be considered as legitimately allied to the art of medicine; but so many observations elucidative of that science occur in it, that we cannot silently pass it over. Mr. H. Cline having addressed the public on the modern method of improving the breed of animals, Mr. Hunt controverts his opinions; and, in this work, enquires into the first principles of the breeding system, asserting, that they are not influenced by consanguinity; enters into an exposition of the theoretical and practical part of the breeding system; the general improvement of animal nature, and its progress toward perfection. Mr. Hunt thinks Mr. H. Cline's employment as an anatomist, is unfavourable to an investigation of the properties of living animals.

"To search for the vital powers when life is gone, must prove a vain pursuit. But he who daily views the whole machine in action, who watches all its motions, and estimates its powers, and, when the balance sinks beneath the healthy standard, with cautious hand adds a few grains

 of

* Anatomical Reflections on the Form of Animals, and the New Opinions of H. Cline, Esq. 8vo. Lond. 1806, pp. 96.

of salutary influence to turn the scale, will certainly know more about the vital powers than he who, when the spring of life has failed, attempts to pry into the worn out mass, and with unnatural conjectures vainly tries to fill the empty space, and presumes to shew how nature moved the work with sympathetic sway." Mr. Hunt contends from his local advantages, that he has the chance of understanding the subject better than Mr. Cline; and that an attention to the *viva vox naturæ*, is of more importance than any anatomical investigation made upon the dead body.

A work of much ingenuity,* under the denomination of the "Anatomy of Expression," from the pen of Mr. Charles Bell, though it is not strictly professional, cannot be overlooked. It is the wish of the author to "demonstrate the importance and uses of anatomy; to multiply the motives for cultivating the science; and to shew how various and how interesting are the deductions which may be drawn from the contemplation of the animal frame."—He divides his work into six Essays, in which he treats of the use of anatomy to the painter, in drawing from the academy figure; of the skull and form of the head; of the muscles of the face in man, and in animals; of the expression of passion, as illustrated by a comparison of the muscles in man and in animals. Of the muscles peculiar to man, and their effects in bestowing human expression; idea of a living principle in the expression of emotion, and of the action of the muscles of the face, as expressive of passion; of the economy of the living body as it relates to expression and character in painting. A number of spirited sketches accompany and illustrate each Essay.

The doctrine of fever has so often been the Ulyssean bow with which every one has been desirous to try his strength, and the occurrence of febrile derangement is a disease so common, that we are somewhat surprized to find but few publications upon it, in the year 1806. Dr. Thomas Sutton has given a clinical account † of a remittent fever of frequent occurrence among the military in this climate; and Dr. H. S. Jackson has published Observations on the Gibraltar Epidemic. ‡ In the first of these, the

* Essays on the Anatomy of Expression in Painting. 4to. pp. 186, many plates.

† Practical Observations on a Remittent Fever, frequently occurring amongst the Troops in this climate, 8vo. Lond. 1806, pp. 42.

‡ Observations, &c. on the Epidemic Disease which lately prevailed at Gibraltar, intended to illustrate the Nature of contagious Diseases in general. Part 1st, 8vo. 1806.

the disease becomes interesting from its resemblance to typhus, and because the stimulating plan, indiscriminately adopted in that fever, is highly injurious to this. With symptoms of extreme debility, are always associated, either violent visceral inflammation, or great local conjestion in some important organ. There seems much reason to admit, that the disease was spread by contagion; the medical attendants on the sick, and servants attached to the regimental hospitals, having very rarely escaped, and in some instances, the whole of them have taken the disease. The remedy chiefly relied on was blood-letting. In one case, 80 ounces were drawn with the best effect; in others, a single evacuation of 30 ounces proved a cure. The pamphlet is written with great care, and with a degree of compression, that renders it impossible to convey the meaning in fewer words than has been done by the author.

The epidemic disease which excited such alarm, and was so fatal in the fortress of Gibraltar, has been theoretically examined, and elucidated in the 2d of them, by Dr. Jackson. This writer contends that this fever is not contagious, but that it is merely symptomatic of a topical affection of the brain. The volume is somewhat desultory, treating on many subjects, not very closely connected with the title page. The monthly Journals, and the News-paper Reports are the sources from whence Dr. J. appears to have gained his information.

In the British Islands, pulmonic complaints, and particularly phthisis pulmonalis, are acknowledged to be of more frequent occurrence, and to have a more fatal termination, than elsewhere. Many intelligent practitioners, who have had the means of extensive information, will reluctantly admit that any case of phthisis, when so far advanced, as unequivocally to mark its genuine character, is ever cured.

If the disease is not thus invariably fatal, still its termition is so commonly unfavourable, under every mode of treatment hitherto applied to it, that humanity seizes with avidity on a promise to improve the remedial process. In this view, the publication of Dr. Bourne* will excite an interest proportioned to the wishes of the Faculty, rather than coincident with their judgment. In the *uva ursi*, Dr. B. hopes he has discovered a substance that will check the

---

* Cases of Pulmonary Consumption treated by *Uva Ursi*; to which are added, some practical Observations, 8vo. Lond. 1806. pp. 293.

the progress, and perhaps cure, this generally fatal distemper. Having observed a case of apparent ulceration of the bladder, accompanied with hectic and emaciation, cured by the *arbutus uva ursi*, Dr. B. was led, by analogy, to apply it as a remedy, in ulcerated lungs. De Haen, Gerhard, Quer, Girardi, and Buchoz, who originally recommended this plant in nephritic cases, about 1760, gave it powdered, in doses from 20 to 60 grains. In such doses our author found it unsuccessful in complaints of the urinary organs, but was more fortunate when he employed Dr. Ferriar's formula.* Sixteen cases of apparent phthisis are here related, as treated by the above remedy. Of these, three died; two while taking the medicine, and one the succeeding winter; nine recovered, and four were apparently relieved. Without exciting doubts as to the real nature of the detailed cases, it will hardly be a question, whether bark and opium, with which it was conjoined, are powerful means of removing their morbid actions; and a suspicion that *uva ursi* is the least active of the three, will scarcely be considered a scepticism. In a disease where every other effort has failed, we do not feel authorized to reject a new remedy on any reasoning *a priori*, but are called upon to give it a full and fair trial. Dr. Bourne is of opinion that the *uva ursi* will frequently fail from the decayed or imperfect state in which it is generally found in the shops. The green leaves should alone be selected and picked from the twigs, and dried by a moderate exposure to heat. The powder, when properly prepared, is of a light brown colour, with a shade of greenish yellow, and has nearly the smell of good grass hay, as cut from the rick. The taste is at first smartly astringent, or bitterish, but by degrees softens into the flavour of liquorice. These are the criteria of its goodness; and when thus selected and prepared, we sincerely hope, though we cannot repress our doubts, that it will be found to answer the Aldrichian Professor's expectations

The preceding Treatise has pointed out a new medicine for phthisis, and is properly confined to a statement of facts, in evidence of the powers of the proposed remedy. Dr. John Reid has published a volume,† which goes more deeply

* Ten grains of the *uva ursi*, rather more than that quantity of bark, and half a grain of opium, given three times a day, was the formula used by Dr. Bourne, in the cases here given.

† A Treatise on the Origin, Progress, and Treatment of Consumption. 8vo. Lond. 1806. pp. 317.

deeply into the subject, and examines this frequent and fatal disease in all its phenomena, bearings, and connections, with a spirit of investigation and research that does much credit to his industry and talents. On a disease of so frequent occurrence as phthisis pulmonalis, that out of the vast population of the British islands, scarcely an individual is to be found who does not fear its ravages either in himself, his friends, or his family; a treatise, written in the manner, and with the views of Dr. Reid's, must, we suppose, be peculiarly acceptable. With the liberal spirit that characterizes the scientific physician of the present day, this author has labored to render his disquisitions not only interesting to the professional student, but open to general observation; he has delineated a portrait, the prominent character of which will be universally understood, while the minute lines and shades of expression remain for detection by the skill of the artist.

In the arrangement of the work, the author, after a general description of the anatomy and physiology of the respiratory organs, delineates the characters of those affections of the lungs, which are considered as præcursors of phthisis. He then considers the nature of consumptive disposition, the mode by which such disposition is best obviated, and concludes by tracing the steps of the malady during its more ordinary and insidious progress, when the preceding affections of the lungs are scarcely marked by characters of sufficient decision to authorize the affirmation of their existence.

A summary view of the 13 chapters into which this treatise is divided, may induce the professional student or the inquirer into general science, to a more minute examination of its contents; where they will find a very full delineation of our state of knowledge on the physiology and diseases of the respiring organs: and though they may not agree in every point with the author, they will not hesitate to acknowledge that he has industriously collected, and arranged with ability, an extensive mass of interesting and useful materials.

The 1st chapter is introductory, and gives a view of the ancient and modern opinions on pulmonary ulcer, an outline of the Brunonian theory, and a summary of chemical physiology; to this succeeds the anatomy of the respiratory organs; the composition of the atmosphere, in which the notions of Aristotle, Lord Bacon, and Mayow are given; the discoveries of Black, Cavendish, Scheele, Priestly, and Lavoisier are stated; and a demonstration of the component ponent parts of atmospheric air, by analysis and synthesis; general physiology of respiration, illustrations of Black's discoveries, Crawford's theory of animal heat, &c. Cutaneous perspiration; hæmoptysis; catarrh; pneumonia, and the utility of digitalis, especially as a preventive of phthisis; tubercles, and the different manners in which the lungs become ulcerated; disposing and immediately exciting causes of consumption; means of destroying the consumptive disposition; history and treatment of the disease. The concluding chapter contains observations on disorders resembling pulmonary consumption in external character.

Since the expedition into Egypt, there has appeared in Europe, an Ophthalmia accompanied with peculiar circumstances of virulence, and spreading at times so rapidly, and with phenomena so marked, that little doubt remains of its being contagious. The British military has suffered materially by it; and many soldiers, in the prime of life, have become absolutely blind. In the year 1802, Dr. Edmonston published an account of an ophthalmia which appeared in the 2d regiment of Argyleshire Fencibles. In this treatise, the contagious nature of the disease was first publicly maintained; and it was clearly traced to an Egyptian origin. There were circumstances in the history of this distemper so novel, and so interesting, that Dr. E. has this year pursued the inquiry in a general treatise on ophthalmia,* in which the question of the contagious nature of that disease is very ably investigated. In the prefaratory part of this Essay, such a variety of facts are brought into one point of view, that no doubt can remain of the existence of a contagious principle disseminating the Egyptian ophthalmia. It is a curious fact in the history of diseases, that the causes producing this ophthalmia, did not operate in Egypt until the country became subject to the dominion of Barbarians.

Dr. Edmonston does not consider the Egyptian ophthalmia essentially differing from other severe varieties of the disease. It arises from external causes; but when numbers are affected, contagion is the result;† and the disease is rapidly

---

* Observations on the Varieties and Consequences of Ophthalmia, with a preliminary Enquiry into its contagious Nature. 8vo. Edin. 1806. pp. 319.

† This assertion has always appeared to us obscure and doubtful. A disease of a simple nature occurs, numbers become affected, and by this accumulation of morbid atoms, contagion of a specific nature is generated. Does not this militate against an admitted law of Nature, and partake of the rapidly disseminated, although the remote causes no longer operate. If this writer is correct, and the laws of other contagions support his opinion, this new and formidable disease may, without much difficulty, be exterminated. The sphere of the ophthalmic contagion is very limited, and though it may act to a short extent* by the medium of the atmosphere, yet it is stated, in almost all the instances which have occurred † among the military, and to that part of society it is yet nearly confined; it was produced by an actual and direct application of the virus from diseased to sound eyes. If its sphere of action is thus confined, and there is every reason to believe this is the fact, how easy to stop its progress, by immediately separating the healthy from the diseased. A religious observance of this, would effectually confine it to a few points, and these, by the same observance, might be daily diminished. At present, it does not sporadically arise in this country; ‡ but if care is not taken to check its progress, it will spread so deep among its population, as to be taken for a native. The foreign invader is yet clearly distinguished; while this distinction remains, let every effort be made to destroy it.

The history of the progress of Egyptian Ophthalmia, will

---

the exploded doctrine of equivocal generation? Does the small-pox, or rubeola, ever arise in this manner? Does pestis. But typhus, in all its varieties, is said to be thus produced. Is it not more probable, as well as more consonant to the simplicity and immutability of the laws of Nature, to admit that all contagions are *sui generis;* and that variola, rubeola, pestis, syphilis, contagious ophthalmia, typhus, psora, &c. all arise from their own proper semina?

* The influence of this contagion, operating through the medium of the atmosphere, does not, in ordinary circumstances, *exceed a foot.* Edmonston.

† The influence of this contagion does not appear to extend far from its source of evolution, as, for a considerable time after the first appearance of the disease, it seemed to require almost immediate contact to communicate it to others. It is probable, therefore, from this circumstance, that a great proportion of the cases of this malignant ophthalmia occurring in regiments, are occasioned by the direct application of virus to the eyes. *Those individuals who have attentively guarded against too near an approach, have, in general, compleatly escaped.* Edmonston.

‡ There is another source of infection, different from actual intercourse with the diseased, and which requires the nicest care to destroy. In the form of *fomites*, so characteristic of its principle, this contagion suffers no abatement of virulence. Many persons, whose eyes had withstood the heat and glare of the Egyptian sands, were attacked when at sea on their return home. In this country it has an occasional suspension, as well as sudden re-appearance, only to be accounted for from the contagious principle being lodged in articles of apparel, towels, bedding, &c.

will forcibly point out our danger. "On the return of some of the Egyptian regiments to Malta, the inferior class of courtezans were among its first victims; and by degrees it became very general over the island. Multitudes of soldiers, on their arrival in England from Egypt, laboured under ophthalmia. Some accompanied the regiments on their march through different parts of the country; others were discharged at the peace, and returned to their respective homes. Intimate and frequent intercourse took place between them and persons in perfect health; cleanliness, at any time but little attended to by the lower ranks of society, was here disregarded, for the disease was considered as local and uninfectious. Owing to these circumstances, ophthalmia appeared at the same time in the most distant parts of Britain; and that peculiar modification, denominated Egyptian Ophthalmia, is now familiar to almost every medical practitioner." We hope the disease has not yet spread thus far and wide; and that there are still in Great-Britain, many practitioners who are unacquainted with Egyptian Ophthalmia. But the danger is at our doors, and the faculty is called upon, by every patriotic feeling, by every sense of professional duty, to destroy this foreign enemy. In every stage and form of human society, there are, and must be, gradations of station. At the head of the faculty of physic in this country is placed the London Royal College of Physicians, the protectors of the rights and privileges of the profession, and the guardians of the public health. To that body, on the introduction of any foreign contagion, the appeal is properly made.

Dr. Edmonston's Observations on ophthalmia in general, will be interesting to the medical reader. He divides the disease into two classes: idiopathtic, and symptomatic. Under the 1st, he gives as species, *Ophthalmia mitis et gravis*; psorophthalmia, puriform ophthalmia of new born infants, and intermitting ophthalmia. Under the 2d, he admits only two modifications, as depending on a general morbid state of the system; scrophulous and venereal. Of the syphilitic species, he gives two varieties, acute and chronic. The first variety is sudden in its attack, rapid in its progress, severe in its symptoms, and is accompanied by a copious purulent discharge from the eye. It is connected with gonorrhœa; and an ingenious investigation of the opinion of its arising from a suppression of the gonorrhœal discharge is introduced. The second variety depending on constitutional contamination, is slow in its progress, destroying stroying the organ of vision by insensible degrees; it is accompanied with an irksome sense of itching, most violent in the evening, and has a distinct remission in the morning. Among the observations on the consequences of ophthalmia, a species of opacity of the cornea from simple distention, is now first described. In this case the cornea assumes a milky hue; the individual is subject to temporary blindness without much preceding inflammation; and often with little more than merely a determination to the head. Dr. Barclay, taking a hint from some appearances that occurred while injecting the vessels of the eye, has been enabled satisfactorily to explain the phenomena of this case; and from this explanation, the mode of cure is rationally deduced. To go into an analytical detail of the curative methods laid down by Dr. E. would far exceed our limits, we must therefore be content with recommending the work to serious perusal, and observing, that in the cure, the author is governed by the two distinct stages of the disease, the acute and chronic. The 1st is marked by a sense of heat, acute pain, intollerance of light, and *a florid colour of the vessels of the conjunctiva.* The 2d is characterized by a sense of weight, obscure pain, and turgescence of the vessels, which in this state *are of a dark purple hue.* Early in the active stage, topical bleeding, especially by scarifying the globe of the eye, is of the greatest utility; general bleeding, unless the quantity taken is immensely great, scarcely diminishes the local inflammation.*

On the diseases of the eye, English medical literature has received a valuable addition by Mr. Briggs's translation of Professor Scarpa's Practical Observations.† This work of the celebrated professor of anatomy at the university of Pavia, treats of the puriform discharge from the palpebræ, and of fistula lacrhymalis, of the hordeolum (stye), incysted tumours of the eye-lids, the trichiasis, relaxation of the upper eye-lid, eversion of the eye-lids, ophthalmia in all its varietes, opacity of the cornea following chronic ophthalmia, albugo and leucoma, ulcers of

* Mr. Shaw, of Liverpool, found a mucilage of quince seeds, dropped into the eye, to give instant relief from the intolerable pricking sensation accompanying Egyptian ophthalmia.

† Practical Observations on the Principal Diseases of the Eye, illustrated with Cases. Translated from the Italian of Antonio Scarpa, by James Briggs, Surgeon, &c. 8vo. Lond. 1806.

of the cornea, pterigium, encanthis, hypopyum, *procidentia iridis,* cataract, artificial pupil, staphycoma, dropsy of the eye, amaurosis and hemeralopia, calculous concretions of the eye. Did our limits permit, we could dwell with satisfaction on the extensive practical information, the ingenious observations, and the scientific views that this volume presents; and to which the translator has done ample justice.

Mr. Reid, surgeon to the 1st battal. of the 89th regiment, has given a history * of an infectious ophthalmia that appeared in that corps. Copious bleeding, tepid bathing, and the topical application of the bread poultice, were the most effectual modes of cure. Blistering was always injurious.

One of the most serious obstacles to the correct practice of medicine, arises from giving a formidable name to a disease of a comparatively mild nature. Under this mistake, consumption and cancer have frequently been cured. A precise ascertainment of the real character of a disease, is an essential point in establishing what modes of cure are fitted to it; what articles of the materia medica are to be depended on, and what to be rejected. The facility with which Mr. Carmichael † cures cancer with the carbonate of iron, raises a doubt of his cases being genuine Carcinoma. But even if they were not truly this formidable disease, they were evidently of a nature to be cured with difficulty; and the faculty is indebted to him for a method, which has been speedily beneficial. With the ingenious Dr. Adams, Mr. Carmichael maintains the independant life of Cancer. And this opinion has led him, by a remote analogy, to the use of a new remedy. Knowing that iron was destructive of intestinal vermes, it occurred to him that it might be equally effectual in destroying other parasitical animals, among which he classed the carcinomatous. The carbonate of iron was employed internally, to invigorate the constitution, and improve the general health; it was applied in fine powder to the ulcers, or sometimes made into an ointment: a lotion of a solution of sulphate of iron was at other times used, while the carbonate was given in large and repeated doses.

Dr.

* An Essay on Ophthalmia: containing a History of that Disease, as it appeared in the 1st Battalion of the 89th Regiment, with some Observations on its Causes and Symptoms. Also the Medical Treatment, &c. 8vo. Portsea. pp. 34.

† An Essay on the Effects of Carbonate of Iron upon Cancer, with an Inquiry into the Nature of that Disease. By Richard Carmichael, Surgeon, 8vo. Dublin, 1806. pp. 116.

Dr. Fraser's Essay* on Epilepsy, excites doubts similar to those which were suggested by a perusal of the preceding work. The frequent failure, however, of every endeavour to cure a disease of so common occurrence as Epilepsy, gives an interest to whatever is written on the subject; and if Dr. Fraser has not materially added, either to our knowledge of the pathology, or to the curative indications, he has produced some facts that demand a further trial of a substance, which superstition originally brought into the materia medica.

At various periods, from the time of Pliny, misletoe has been considered as a specific in epilepsy; but from its slight medicinal properties, if it is not altogether inert, much of its credit must have arisen from some mental impression, operating on a disease often consisting of irregular actions of the nervous system, independant of organic derangement. Many instances have occurred of vivid impressions on the mind producing a suspension of the paroxysms, and finally, a removal of the disease. A lady, in the prime of life, robust habit, the mother of several children, had, for four years, been afflicted with epilepsy in a violent degree. The paroxysms returned three or four times a week, continued some hours, and left the patient in a state of stupor. Advice was sought in every quarter, and medicines of every indication were given with determined perseverance; but the violence of the disease was not abated. Under these circumstances, and when she had abandoned the use of medicine, her second daughter was accidentally burned to death; from that moment the epileptic paroxysm returned no more. This pamphlet contains eleven cases, in which the *Viscus Quercinus* was presumed to have been of service. Of these eleven cases, nine were radically cured, one was fatal, and one received no benefit, p. 89.

Diseases of the digesting organs, and of the abdominal viscera in general, have received an attention during the year 1806, that cannot fail to meliorate the practice in the numerous train of morbid actions connected with, or arising from, parts of the animal machine, so powerful in their influence over its whole fabric, and so arbitrary in directing the operation of all its functions.

We shall notice first a small tract, by the late M. Dau-

* On Epilepsy, and the Use of the Viscus Quercinus in the cure of that Disease. 8vo. Lond. 1806. pp. 96.

benton,* translated from the French by Dr. Buchan. The object of this memoir is to point out the utility of small doses of ipecacuanha, in those debilities of the stomach, and indigestions, which occur at the approach of old age. M. Daubenton, who was the associate of M. Buffon, in all his splendid literary labours, has, in this little memoir, given a number of ingenious observations on the actions of the stomach; and is peculiarly clear and decisive in his proofs of the efficacy of the remedy recommended.

A Treatise on the Diseases of the Stomach,† by Dr. D. A. Stone, abounds with practical information, and gives an extensive view of a train of morbid affections of so frequent occurrence that few have been enough fortunate to escape them altogether; and of which the treatment has, notwithstanding, been frequently erroneous and often absurd. Dr. Stone divides his work into three parts; in the 1st, he gives the anatomy and physiology of the stomach and intestines; in the 2d, the history of their diseases; and in the 3d, the treatment of those diseases.

To a brief description of the structure of the stomach and intestines, and a sketch of the physiology of those parts, containing observations on the experiments of Professors Dumas and Spallanzani; those of Prof. Werner of Tubingen, on chyme; and Scheele, Parmentier and Deyeux with acids and milk; a history of the diseases of the stomach succeed; a vitiated state of the fluids of the stomach; marasmus; repletion of the stomach; poisons; the state of the stomach and abdominal viscera after a residence in hot climates; the state of those organs produced by hard drinking; pyrosis; hæmorrhagy from the stomach and malœna; hypochondriasis and sick head-ach; flatulency, tympanites, cardialgia, &c. are examined with a view to practical utility. In the 3d part, the chapter on the use of emetics, deserves a serious attention. He examines the authorities for, and the objections to, the use of emetics; sea sickness; fallacies, respecting the use of emetics, arising from spontaneous vomiting; objections to violent emetics, and the danger of antimonial emetics for children;

---

* Observations on Indigestion: in which is satisfactorily shewn the efficacy of Ipecacuan. in relieving this, as well as its connected train of complaints peculiar to the decline of life. 8vo. Lond. 1806. pp. 24.

† A Practical Treatise on the Diseases of the Stomach, and of Digestion; including the history and treatment of those affections of the liver, and digestive organs, which occur in persons who return from the East and West Indies. 8vo. Lond. 1806. pp. 291.

dren; the proper mode of exhibiting various emetics; their use in poison, and the state resembling apoplexy, and where there is indigestible matter in the stomach; in scarlatina, small-pox, and croup; in hooping cough, pulmonary diseases, hæmorrhagy, epilepsy, and to promote absorption. There are few practitioners, who have not to lament the consequence of this violent inverted action of the stomach. In some instances, we have seen death ensue from the first operation of emetics; in others, an incurable debility of the stomach has been the consequence, oppressing life, and embittering all its enjoyments for years. Dr. Stone has been well employed in endeavouring to establish a rational use of a medicine capable of doing some good, or much mischief; and in correcting "its common and capricious use.*"

The marked characteristic of Dr. Pemberton's Treatise on the Diseases of the Abdominal Viscera,† is that of its being the result entirely of his *own* practice, or having arisen out of his *own* experience in the practice of others. A work raised on this foundation must possess some originality; and its chance of adding to our funds of medical knowledge, will be proportioned to the faithfulness with which this promise is kept in view. This production is elegantly printed by Bulmer, and in eleven chapters, details the history and treatment of acute and chronic inflammation of the peritonæum, and hydatids in the omentum; acute and chronic inflammation of the liver; diseases of the gall-bladder, jaundice occasioned by gall-stones, and the passage of biliary concretions, with a plate of the *vesica fellis*; diseases of the pancreas; inflammation and indolent swelling of the spleen; symptoms of disease in the kidneys; observations on emaciation; different calculi of the bladder; analysis of a calculus from the human pancreas, and the passage of a calculus along the ureter; pain in the stomach when empty, pyrosis, affinity between pyrosis and diabetes, pain in

 the

* In the narcotic poisons, particularly opium and hyosciamus, Dr S. has found ammonia strikingly serviceable. Where opium has been swallowed, and an alarming stupor has continued for many hours, *a spoonful of a strong solution of ammonia has awakened the patient, and enabled him by words to express the benefit he has received from it.* In a case mentioned, p. 76, draughts of ammonia quickly overcame the symptoms produced by hyosciamus. p. 195.

† A Practical Treatise on various Diseases of the Abdominal Viscera. 8vo. Lond. 1806. pp. 197. Two plates.

the stomach when full, vomiting in consequence of nausea unattended by pain, formation of acid in the stomach, organic diseases of the stomach, a continued state of vomiting and hæmorrage from the stomach; diseases of the intestines, cholera morbus, dysentery, diarrhœa, colica pictonum, and febris infantum remittens; inflammation of the peritonæal coat of the intestines; inflammation of the mucous membrane of the intestines; disease of the mesenteric glands.

The ambiguity of the symptoms of great and even fatal disease going on in the abdominal fystem, renders every effort to elucidate, either directly or indirectly, this obscure subject, deserving the serious attention of the faculty. Among the investigations of Dr. Pemberton, we have been particularly interested by his observations on the decrease or non-decrease of the bulk of the body, as indicatory of the seat of certain local chronic affections. The glands of the body are, either those which secrete a fluid from the blood for the use of the system, or those which secrete a fluid to be discharged from it. The first are called by Dr. P. *glands of supply*; the second, *glands of waste*.* If disease is seated in the former, rapid emaciation takes place; if in the latter, the bulk of the body is diminished. In a diseased state of the mesenteric glands there is great emaciation; in a scrophulous affection of the breast, none at all; in ulceration of the small intestines, the body is wasted; in schirrus rectum it is not. In a disease of the gall-bladder the bulk of the body is rapidly diminished; in affections of the urinary bladder scarcely any diminution is perceived. In abscess of the liver, the body becomes emaciated; in abscess of the kidney, it preserves its size.† A recurrence to the functions of these two systems of glands will furnish a strong argument, *a priori*, in support of Dr. Pemberton's opinion; and should future observation confirm the fact, the profession will be indebted to his treatise for a guide through this labyrinth of disease.‡

Mr.

* "The glands which secrete a fluid to be employed in the system, as well as the glands of direct supply, may be considered, the liver, the pancreas, the mesenteric glands, perhaps the stomach and small intestines; and the glands of waste are the kidneys, breasts, exhalent arteries, and the large intestines."

† "The presence of pain in a part will sufficiently point out the seat of the disease. It is only in chronical complaints, where the morbid changes are so gradual as to produce no pain, that these observations will particularly apply; and it is in difficulties of this nature that we stand in need of every help." p. 86.

‡ As a view to practical utility, it is hoped, will be a marked trait in this Historical Retrospect, we cannot refuse to notice Dr. Pemberton's method

Mr. Abernethy, as well-known for his philosophical views of the principles of his art, as for his great skill in operative surgery, has this year published Observations on those disorders of the Digestive Organs which accompany local Diseases.* Having been led to pay attention to the digestive organs, and their connexion with other diseases, in the relation of cause and effect; the importance of the investigation gradually increased in his estimation. He perceived that local diseases disturb the functions of the digestive organs; and, conversely, a deranged state of those organs, either occurring in consequence of such sympathy, or existing primarily as an original disease, materially affect the progress of local complaints. It is the object of this volume, to establish the foregoing principle, by a relation of numerous facts; to elucidate its mode of operation, by reasoning on those facts; and to confirm the practice by a detail of successful treatment. In addition to the investigation of disorder in the digestive organs, occasioned by local affections, or, as retarding the cure of topical disease, will be found an elaborate view of the functions of the chylopoetic viscera, and a detail of morbid appearances that have often been mistaken for syphilis; but which have uniformly been cured by general means applied to the organs of digestion. Some cases and observations on diseases of the urethra, particularly that part which is surrounded by the prostate gland; and remarks on the treatment of one species of *nævi materni,* close the volume.

To those who are accustomed to look into the events that occasionally agitate the medical world, the case of Dr. Greenfield will be familiar. Upon this unfortunate man a degree of obloquy was cast, on account of his use of cantharides internally, and his Treatise on that subject, which neither his conduct nor his book deserved. Since that time, we do not recollect any express work on the internal use of the cantharis, before the appearance of

 Mr.

method of treating paralysis of the hands. The principle of the practice is to place, by some mechanical contrivance, the paralytic muscles in a state to resume their lost action. This is effected by placing the hand and fore-arm upon a splint, so as to take off the weight of the hand from the diseased muscles. A plate accompanies the description of the method, with instances of its success.

* Surgical Observations, Part the Second, containing an account of the disorders of the health in general, and of the digestive organs in particular, which accompany local diseases, and obstruct their cure, &c. 8vo. Lond. 1806. pp. 245.

Mr. Robertson's "Practical Treatise."* That the *Meloe vesicatorius* possesses active properties, and has the power of exciting extraodinary actions in the animal economy, will not be denied; but whether this acrid insect will afford "a stimulus capable, not merely of producing temporary excitation, but ensuring permanent support, without being followed by the debilitating consequences of some, or the narcotic effects of others;" those, who have seen how suddenly great promises fade into "thin air," will, at least, doubt. Much is due to Mr. Robertson, however, for his researches, which, if they do not possess in any extraordinary degree the *suaviter in modo,* have yet been pursued with intelligent industry. The three sections into which the work is divided, treat of the use of this insect, in gleet, in leucorrhœa, and in obstinate "*sore.*" Perhaps, Mr. R. is too sanguine when he asserts, that the cantharis, taken internally, "operates as an agreeable and universal stimulus, infusing not only action, but the power of maintaining action, dispelling atonia, and re-establishing health."

A Treatise on the Gravid Uterus, published a few years since, gave Mr. Burns considerable credit with the faculty; and in some Observations published† this year, closely connected with the same subject, he has supplied a deficient link in the chain of English medical literature. In the treatise on abortion, the general principles, the prevention and treatment of the disease, are perspicuously explained under the formation of the ovum, the causes that give rise to abortion, &c. Some new and interesting speculations on the formation of the ovum, occupy the first part of the work; to this succeeds a view of the phenomena of abortion. Mr. Burns, in his dissertation on inflammation, has an hypothetical notion, that when the action of any part is increased, the energy of other parts must be proportionally diminished. On this hypothesis he endeavours to explain the causes of some abortions. There being increased actions of the uterus in gestation, requiring an increased quantity of energy to support it; the system is put, *pro tempore,* into an artificial state, and obliged either to form more energy, which cannot be so easily

* A Practical Treatise on the Powers of Cantharides, when used internally, demonstrated by experiment and observation. 8vo. Edin. 1806.

† Observations on Abortion, containing an account of the manner in which it is accomplished, the causes which produced it, and the method of preventing or treating it, 8vo. Lond. 1806. pp. 139.

easily done, or to spend less on some other part. Thus the functions of nutrition, or the action by which organic matter is deposited in the room of that which is absorbed, often yields or is lessened, and the person becomes emaciated, or the stomach has its actions diminished, or the bowels becoming inert, produce costiveness and inflation. If no part gives way, and no more energy than usual is formed, he argues that gestation cannot go on, or goes on imperfectly.* Hence, some women have abortion induced by being too vigorous. In this species, which Mr. B—— considers as the result of too firm action, half a grain of digitalis, and one-eighth of a grain of tartrite of antimony given every night, is serviceable, by diminishing the action of the digestive organs. Digitalis is the favoured remedy of this writer, and is given under every circumstance of abortion and flooding, where it is the indication to diminish vascular organic action.

The subject that has, during the year 1806, excited the most lively interest; and which, indeed, has most deserved a serious and dispassionate investigation, has been the History of the Cow-Pock; and an Inquiry into the reasonable security Vaccination affords against Variolous Infection. The practice of vaccination has now taken so wide a range, and such multitudes throughout the world have adopted it as a security against the small-pox, that few persons will be found so far removed from civilized life, as not to have some curiosity respecting the opinions that agitate society on this subject.

When inoculation for the small-pox was introduced into England, and for a few years after that event, from motives now not ascertainable, several persons of no mean talents, raised up a cry against it; and endeavoured by arts equally unfair and uncandid, as far removed from rational inquiry, as from honourable investigation, to stop its progress. This was attempted to be done, not by reasoning, but by exciting prejudices, by false histories, by popular abuse, and by appeals to conscience. At the head of this combination were some of the Clergy, particularly the Rev. Mr. Massey, and M. de la Faye. What these gentlemen were to inoculated small-pox, Dr. Rowley

 was,

* We have a certain quantity of action present in the system at large, and properly distributed among the organs, forms an equilibrium of action; but if any organ act in an over degree, another which is connected with it, will have its action lessened, and *vice versa*. Burns.

was, and Dr. Moseley now is, to the vaccine disease. Placards, handbills, poetry and prose, in every form and feature, have been called into action; while dispassionate reasoning and cool investigation have been rejected. To retard the progress of a most fatal and destructive operation, according to the assertion of its opposers, and which gives no present security, entails on its victims future diseases the most loathsome and horrid in their external form, and perfectly novel in their symptoms; intemperate language, exaggeration of facts, and aspersion of character, seem to have been understood as the most certain and effectual means. The leader of the phalanx thus employed, has paid the debt of nature. Some learning, great activity of mind, strenuous application to business, in him were combined with blind prejudice, bigotted zeal, and a determination to rule the public opinion without a nice regard to the means employed. The discovery of Jenner was opposed by Dr. Rowley with a pertinacity of abuse, that must lead to an opinion, among those who judge from the mode of attack, that he had a personal enmity to the man, rather than a conviction of the ill consequences that might arise to society from the employment of the vaccine virus. There is no arriving at the private motives that might actuate Dr. Rowley in this controversy; but his public conduct is open to reproof, and the chastisement of criticism has fallen upon it: though, perhaps, occasionally itself manifesting a similar deviation from cool, rational, and dignified scientific investigation.* A physician of much ingenuity, and great quickness of parts, became the coadjutor of Dr. Rowley in the vaccine controversy. That Dr. Moseley should adopt opinions different from others on the subject of the vaccine disease, is in no wise reprehensible; by the opposition of sentiment, and the collision of inquiry, truth is elicited. But when a man of talent, and who has heretofore deserved well of the

* Dr. G. Pearson has observed (Phil. Mag. June 1806, p. 93.) that the publications in favor of vaccination are written, for the most part, with the spirit of animosity, and not of investigation; and that the adverse writers seem not at all anxious for any thing but to collect and publish inaccurate accounts of failures, without a due regard to the laws of evidence. Neither party seems to be aware that vaccination may leave one out of a thousand still liable to the small-pox, and yet finally effect the grand object in view, the extermination of the small-pox, by substituting an affection that is not, in one case in a hundred, worth calling a disease, and produces death scarcely once in ten thousand cases; for small-pox inoculation, which produces a severe disease in one case of twenty-five, and death in one of one hundred and fifty or two hundred.

the public, and of science, condescends to gross exaggerations, and to arts the vulgar and the unlettered understand and practice with an adroitness equal to himself; we lament the degradation of mental powers. Mr. Birch, and Mr. Goldson, with a few others of meaner note, may be numbered among the opposers of vaccination. These gentlemen are, however, to be distinguished from the herd of anti-vaccinators, by a greater degree of candor and moderation in their opposition.

Since the death of Dr. Rowley,* the anti-vaccinarian spirit has languished. In the year 1806, very few publications have appeared to oppose the use of vaccine inoculation. A second edition of Dr. Moseley's "Commentaries," † Mr. Birch's "Serious Appeal," ‡ Mr. Lipscomb's "Cow-pox exploded," || and a second edition of Dr. Squirrel's Observations, § are, we believe, the only distinct and separate publications, on this subject, that have issued from the British press in this year.

Of a second edition of Dr. Moseley's Work, it will not be expected that a minute account should be given; it has been long before the public, and its peculiarities are well known.¶ In Mr. Birch's "Serious Reasons," are there any new facts brought forward? Many are, however, promised, and Mr. Birch speaks of being in possession of a great body of evidence; but until it is publicly produced, its credibility and weight are uncertain. This author labours to prove that vaccination has been often fatal, that it has introduced new disorders into the system, and that it

---

* Dr. Rowley died on the 17th of March, 1806.

† Commentaries on the *Lues Bovilla*, or Cow-pox; second edition, 8vo. Lond. 1806. pp. 260.

‡ Serious Reasons for uniformly objecting to the Practice of Vaccination; in answer to the Report of the Jennerian Society. 8vo. Lond. 1805.

|| Cow-pox Exploded; or the inconsistences, absurdities, and falsehoods of some of its defenders exposed. 8vo. Lond. 1806. pp. 57.

§ "Observations on the pernicious Consequences of Cow-pox Inoculation; containing many well authenticated instances, proving its insecurity against Small-pox: also Remarks on the Advantages of Small-pox Inoculation. Second edition, small 8vo. Lond. 1806. pp. 74."

¶ The Preface is, however, new, and the Doctor says in it, that future ages will read with wonder the history of the cow-pox credulity of this nation. He calls the vaccine disease a cacodæmon, and those who associate to support it, a diabolical conspiracy; and adds, from this cow-pox medley of weak philosophers and strong fools, the world will form some estimate of the state of physic in England. He consoles himself with the idea that the spring, summer, and autumn of 1805, decided the fate of cow-pox in England. The monster expires on its *natale solum*, he exclaims with exultation.

it is not a security against the small-pox. He also complains that the supporters of the practice have used unfair means in defending it; that the royal patronage and authority of parliament have been used to an unwarranted degree; that the command to the army and navy has been adduced, not as a means of facilitating the experiment, but as a proof of the triumph of the cause; and that the monopoly of the press, and the freedom of the post-office, have been employed to circulate the assertions of the friends of vaccination, and to suppress the arguments of their opponents. Observations on the Report of the Royal Jennerian Society, and a Critique on Mr. James Moore's "Reply," occupy a principal part of this pamphlet. The "Observations," by Dr. Squirrel, are addressed to the King; and the object of the author is to prove, that the cow-pox, as originating from the grease in the horse, is a modification of scrophula; that it introduces that disease into the system, and is not a security against the small-pox. At page 24, he gives a comparative view of the cow-pox and small-pox inoculation, which, he says, "must convince every dispassionate person, that the former is a malignant disease, undermining the constitution, and subjecting the human body to many troublesome and lingering disorders; and ought, therefore, to be exploded out of practice, or it will soon destroy the health of the human race."* Mr. Lipscomb having pledged himself to the public † to reply to every argument which might be brought forward by Drs. Jenner, Pearson, Lettsom, Adams, or

* 1. The cow-pox inoculation produces malignant effects, vitiates the blood and other juices.

The small-pox inoculation produces no ill consequences whatever.

2. The cow-pox produces very ill health to children.

The small-pox inoculation improves the health and constitution.

3. The cow-pox matter is taken from an animal diseased, and is of a specific scrophulous kind.

The small-pox matter is taken from a healthy subject, and produces no disease whatever but the one for which it was intended.

4. The cow-pox was introduced into this country in 1798, since which time experience has proved that it has produced many bad consequences.

The small-pox inoculation has been practiced nearly 100 years in this country, and no ill effects can with truth be attributed to it.

5. The cow-pox is a disease unnatural to the human constitution. Providence never intended that it should affect, *or pester*, the human race; consequently, vaccination must be repugnant to nature.

The small-pox is a natural disease, which was sent to us by providence, and has afflicted mankind according to the law of nature.

† Dissertation on the Failure and Mischiefs of the Cow-pox.

or Thornton, fulfils, as he says, this promise by the publication of "Cow-pox exploded," with which he hopes to close the controversy. This pamphlet, which the author's self-complacency leads him to imagine is so powerful in facts, and so convincing in arguments, as to render all further contest futile, is merely an attack on Dr. Thornton's *Vaccina Vindicæ.* It abounds with that species of rhetoric which has so often disgraced the combatants on both sides; and is filled with flippant criticism, and personal invective.

The supporters of the Jennerian Discovery have been actively employed in its defence; and much talent and ingenuity have been exercised in ascertaining the real properties of the cow-pock, and in elucidating its symptoms and history. At the head of the publications on this subject, during the year 1806, we are disposed to place Dr. Willan's Treatise.* This quarto volume contains a view of all that has been written of any importance on this subject, arranged with all the clearness and precision we expect from Dr. Willan, and enriched with remarks which manifest a thorough acquaintance with the disease. This work, consisting of 108 pp. and an appendix of 55 pp. is intended to exhibit the result of a laborious investigation, without reference to controversies. It treats, 1st. of the combined inoculation of the variolous and vaccine fluids. 2dly. on the characteristics and effects of perfect vaccination. 3dly. on imperfect vaccination. 4thly. on variolous eruptions subsequent to vaccination. 5thly. on cutaneous and glandular affections imputed to vaccine inoculation. 6thly. on the chicken-pox and swine-pox. 7thly. on the inoculation of varicella. The Treatise concludes with remarks on a general plan for exterminating the small pox. The appendix contains observations on the cow-pock by Dr. Jenner, and Mr. John Pearson; Report of the progress and present state of vaccination at Liverpool, at Lancaster, Edinburgh, Dublin, and Cambridge; — at Bath, Yarmouth, Newcastle upon Tyne, Manchester, Carlisle, Exeter, Reading, York, Monmouth, Salisbury, and Norwich. Two highly finished coloured engravings shew the variolous pustule and vaccine vesicle contrasted, spurious vaccine vesicle, pustules and scab of the favus, a papulous eruption

* A Treatise on Vaccine Inoculation; to which is added an Account of the Chicken-pox, Swine-pox, and Hives, with coloured engravings. 4to. Lond. 1806.

ruption which sometimes occurs during vaccine inoculation, *area* or ring-worm of the scalp, lenticular, conoidal, and globated varicella.

Dr. Adams has very properly defended Vaccination, by answering * objections with candour and moderation.—Those who are acquainted with Dr. Adams's elaborate Work on Morbid Poisons, will have little doubt of the ingenuity he has shewn in defending a cause in which he is officially engaged, as physician to the Small-pox Hospital. It is a remarkable circumstance, that objections have been no where raised against vaccine inoculation, but in England. Through France, Spain, Italy, Portugal, Germany, Prussia, Russia, and every other part of Europe; through the East Indies, and most other parts of Asia; through Africa and America, even to the savage native tribes, the practice of vaccination has extended, and no objections have arisen. † In this particular it differs from the

* An Answer to all the Objections hitherto made against the Cow-pock. 8vo. Lond. 1806. pp. 37.

† In the year 1800 the late Dr. Woodville, by particular invitation, went over to France for the purpose of introducing vaccination to that country. In 1802, the annual report of the central committee declared its success, and the report of this committee for 1804 asserts that the practice of vaccination is fully established by 100,000 facts. Med. and Phys. Journ. May 1805, p. 419.——In Spain the practice has been received with avidity, and has been followed by the happiest success.——In the summer of 1800, Drs. Marshall and Walker left England for the purpose of introducing the cow-pox to the shores of the Mediterranean. They began at Gibraltar, thence proceeding to Malta, Sicily, and Naples. In the Italian republic alone, in 1802, Dr. Sacco had vaccinated 70,000.——The success in the dominions of the House of Austria, where it was introduced in 1799, is extremely remarkable. *In* 1798, *the average of deaths from the small-pox in Vienna was* 835; *in* 1802, *this average was reduced to* 61; *in* 1803, *to* 27; *and in* 1804, *the actual number who died of the small-pox amounted to two persons only.*——When the Russian court was at Moscow, in October 1801, the vaccine inoculation was first employed on a child, who was afterwards called *Vaccinoff*; since when it has been established throughout the Russian empire.——The King of Prussia was the first crowned head that submitted his own offspring to vaccination.——In the summer of 1802, the vaccine inoculation was employed at Copenhagen, where it became so general, that the bills of mortality for that town, in the succeeding years, returned none as dying of small-pox.——Bohemia, Livonia, and the two Gallicias,* in 1801, adopted vaccination very generally; and in the dominions of the Elector of Salzburg, and in the Dukedom of Mecklenburg, in 1804 and 1805, it was employed to a great extent.——On the 14th of June, 1802, the first person in Hindostan was vaccinated by Dr. Scot; and since that,

* In the two Gallicias, not less than 65,258 persons were vaccinated in six months. Thornton Vac. Vind. 422.

the early history of inoculation for the small-pox. After a short detail of the opposition to small-pox inoculation, with a view to point out the coincidence of conduct between the enemies to that practice and the anti-vaccinators of the present day, the author proceeds to examine the objections brought against cow-pock inoculation. The whole of these objections are arranged under three heads. 1st. That it is no security against the small-pox. 2dly. That it is only a security for a time. 3dly. That it introduces humours into the constitution. It is admitted that some instances have occurred of small-pox after cow-pock, and on those the opinion of its being no security, or only a temporary one, has been founded. Dr. Adams, however, very distinctly shews that this second infection has arisen from one or other of the following circumstances. By 1st. an imperfect vaccination; 2dly. by the constitution being under the influence of some other disease at the time of vaccination;* or, 3dly. by being liable to the small-pox twice. The objection of its introducing humours into the constitution, is proved to apply to inoculated small-pox in a greater degree. Dr. Adams has given a clear unvarnished account, calculated to instruct all classes: plain, but distinct: if it does not afford amusement, it gives information.

The reply † of Mr. James Moore is characterized by liveliness of manner, perspicuity of arrangement, neatness of style, and acuteness of remark. Few pamphlets will be perused with more pleasure by readers of taste than this, notwithstanding it is written on a subject that promises but little amusement. In treating the question, a clear history of Dr. Jenner's Discovery is given, its claim to scientific distinction appropriated, and its practical utility ascertained. The detail is minute without being prolix, and the result of an inquiry into this subject is distinctly pointed out. When the writings of the anti-vaccinists have any claim to serious examination, they are scrutinized with critical acumen: when they are absurd or self-interested, the lash of satire is justly applied to them.

As

---

that, not fewer than 800,000 have undergone the operation.——In the empire of China, a country whose attachment to old customs has such rooted power over the human mind, vaccination has become very general; and a book in the Chinese language has been published on the subject.—In the beginning of 1799, by the exertions of Dr. Waterhouse, and the patronage of the President Jefferson, the cow-pox gained a firm footing in America.

* Med. and Phys. Journ. vol. xii. p. 97.

† A Reply to the Anti-vaccinists, 8vo. Lond. 1806.

As a specimen of the style and manner of Mr. Moore's "Reply," we select the excellent observations on the conduct of the Faculty to each other, and on their reception of the vaccine discovery.

"Could any class of human beings live in peace, it might be expected of those brought up to the church and to medicine. But ecclesiastics can seldom instill into mankind the doctrine of love to each other, without mutual rancour; and physicians as rarely inculcate acts of humanity, without snarling at their brethren. It is strange, yet true, that seamen and soldiers, whose duty it is to wound and destroy, are hardly more given to quarrel, than churchmen and physicians, whose avocation it is to relieve and save. That vaccination should occasion contention, was a thing of course; but this has been carried to unexpected lengths; for both those who approve, and those who disapprove, have accused each other of murdering their patients. It must be owned indeed, that on this occasion, there was superadded to the general tendency of doctors to differ, a particular motive which rarely fails to have that effect on all mankind. Small-pox was the source of no inconsiderable portion of the income of almost every medical practitioner; insomuch, that neither physicians nor surgeons would abandon this disease to the management of the other. The physician claimed it as a contagious fever, and therefore a medical case; but as the surgeon was the inoculator, he did not choose to relinquish the profits of the subsequent treatment. While each was eager for the whole, it was hardly to be expected, that a plan to take it from both would be kindly received by either. Jenner's discovery was a touch-stone, to detect what proportion of selfishness alloyed the human heart. It was calculated to make known, whether the scenes of misery, which medical men are compelled to witness, blunt their feelings. The result has certainly reflected distinguished honour on the faculty; for the plan to exterminate the small-pox, has been zealously adopted by the medical men of every part of the world which it has reached."*

To give a detailed account of every pamphlet which has appeared in 1806, on this subject, would far exceed our limits; we must therefore be content with enumerating the

---

* This must be taken with the exception of Drs. Rowley, Moseley, and Squirrel; Messrs. Birch, Goldson, Lipscomb, &c.

the title of many that deserve a more minute attention. The indefatigable Mr. Ring, besides his extensive animadversions in the Medical Journal, has printed a reply * to Mr. Birch, in which the warmth, if not the asperity of his manner, is strikingly characterized. Mr. Moore has also replied to Mr. Birch. Mr. Jones, surgeon to the Montgomery volunteer legion, has addressed a letter to his patients, in vindication of vaccination.† The Rev. Edward Jenner has printed the Report before the House of Commons.‡ Mr. Blair, in "the Vaccine Contest," has thrown the argument ‖ into the form of dialogue, in which he makes Dr. Rowley, under the name of Dr. Bragwell, sustain his part in the language of his own pamphlet. The Rev. Rowland Hill, in a pamphlet § dedicated to the Duke of Bedford, as president of the Jennerian Society, has rendered essential service to the cause of vaccination, by exposing the unfair conduct of its opponents, and by pointing out, very distinctly, its success in a great number of instances. Mr. Creaser, of Bath, has printed Observations ¶ on the Report of the Committee of the House of Commons; and the late attempts to depreciate vaccine inoculation have been investigated by Mr. Merriman.** With Mr. Dunnings's Detail,†† with Doctor Thornton's Vaccina Vindiciæ,

---

* An Answer to Mr. Birch, containing a Defence of Vaccination. 8vo. Lond. 1806.

† Vaccination Vindicated against Misrepresentation and Calumny. 8vo. Lond. 1806.

‡ The report of the evidence at large, as laid before the Committee of the House of Commons, respecting Dr. Jenner's discovery of Vaccine Inoculation: together with the debate which followed, and some observations on the contravening evidence. 8vo Lond. 1806.

‖ The Vaccine Contest; or mild humanity, religion, and truth, against fierce unfeeling ferocity, over-bearing insolence, mortified pride, false faith, and despiration; being an exact outline of the arguments and interesting facts, adduced by the principal combatants on both sides, respecting Cow-pox Inoculation, &c. 8vo. Lond. 1806.

§ Cow-pock Inoculation vindicated and recommended from matters of fact; by Rowland Hill, A. M. with the Report of the Medical Council of the Royal Jennerian Society. 8vo. Lond. 1806.

¶ Observations on Mr. Pearson's Examination of the Report of the Committee of the House of Commons, concerning Dr. Jenner's Claim for Remuneration. 8vo. Lond. 1806.

** Observations on some late attempts to depreciate the nature and efficacy of vaccine inoculation. 8vo. 1806.

†† A short Detail of some circumstances connected with vaccine inoculation, which lately occurred in the neighbourhood of Plymouth, with Remarks. 8vo. London, 1806. The calm, candid, and rational statements of Mr. Dunning carry considerable weight with them.

ciæ,* and some anonymous essays, †, ‡, ||, we must close our catalogue.

On a subject of such importance to society, either in its success or in its failure, as the VACCINE INOCULATION, candour, truth, humanity, reason, and science, should be united. The little arts that are used to support little causes, should be rejected with disdain; sarcasm and wit should not be heard in the dispute; but rational and dispassionate investigation, in this question of science, should be paramount to every hostile feeling: jealousy, resentment, and envy, should be lost in contemplating the magnitude of the blessings its success will bestow, or in reflecting on the quantum of evil that will fall on the human race from its failure. The legislature of this country, feeling very fully the importance of Dr. Jenner's discovery, has provided for a rational decision of this momentous question, by delegating the trust to the President and Fellows of the Royal College of Physicians.§

With an appearance of injustice to several valuable productions of the year 1806, but, in truth, from having arrived at the extent of our limits, we are compelled only slightly to notice, what under other circumstances we should have dwelt upon with peculiar satisfaction. It is this that obliges us to give merely the titles of such works as

---

* Vaccinæ Vindicia, or a Defence of Vaccination; containing a refutation of the cases, and reasonings on the same, in Dr. Rowley's and Dr. Moseley"s pamphlets against vaccination. 8vo. Lond. 1806, pp. 472. Dr. Thornton has been very properly employed in examining the facts, and in ascertaining the genuineness of the cases produced by the anti-vaccinists. The discovery he has made of false statements, interpolation, and fabrication, as it assists the cause of truth, has our best thanks.

† Arguments relative to the Cow-pox, addressed to Lord Hawksbury, and laid before the Board of Health. By a Physician.

‡ A Letter to Mr. Birch, in answer to his late pamphlet against vaccination; by a Member of the Royal College of Surgeons, 8vo. 1806.

|| Lettsom's Exposition of the Cow-pox. 8vo. 1806.

§ On Wednesday, the 21st of July, 1806, it was moved in the House of Commons, by Lord Henry Petty, "That an humble address be presented to his Majesty, praying, that he will be graciously pleased to direct his Royal College of Physicians to enquire into the state of the Vaccine Inoculation in the united kingdom, and to report their opinion as to the progress it has made, and the causes that have retarded its general adoption." The motion was introduced by the noble mover with an eloquent speech, tracing the history and progress of vaccination; and an animated debate took place, in which Dr. Mathews, member for Hereford, Mr. Wilberforce, Mr. Windham, Mr. Banks, and Mr. Wm. Smith took a part, when the address was agreed to without a dissentient voice.

as Dr. Pinckard's Notes on the West Indies, 3 vol. 8vo.; Dr. Beddoes's Manual of Health; Cases of Excision of carious Joints, by Mr. Parke, of Liverpool, 8vo.; the late Dr. Hamilton's (of Lynn Regis) Letter on the Cause und Treatment of the Gout; the Analysis of the Malvern Waters, and Observations on the Use and Abuse of Mercury, by Dr. Phillip Wilson; Peake's Admonitory Hints on the Use of Sea Bathing; Johnson's Practical Observations on Urinary Gravel and Stone; on Diseases of the Bladder and prostate Gland, and on Strictures of the Urethra, 8vo.; Dr. Jones's Treatise on the Process employed by Nature in suppressing Hæmorrhage, &c.; John Bell's 2d volume of the Principles of Surgery, 4to.; Cox on Insanity, 8vo.; Luxmore's Treatise on Hernia Humoralis; Clark's Essays on the Management of Pregnancy and Labor, 8vo.; and improved editions of Arnold on Insanity; Rollo on Diabetes Mellitus; Duncan's Dispensatory; Heberden's Commentaries; Welldon's Cases in Surgery; Chevalier on Gun-shot Wounds; Butter on the Infantile Remittent Fever; Home and Whately on Strictures of the Urethra; Howard on the Venereal Disease, &c. &c.

Before we close this retrospective sketch of the progression of the medical art in Britain, we cannot refuse to notice the establishment of a society in London, for the relief of persons afflicted with Hernia. In the year 1796 a society was founded in this metropolis for the purpose of gratuitously affording chirurgical assistance and trusses, in cases of Hernia, to persons in indigent circumstances; but from some occurrences, that time has rendered unimportant, its laudable object was in a measure frustrated. The spirit of active benevolence, so characteristic of this country, has founded, however, with similar views, an institution called the New Rupture Society.——The address of this Society to the public, contains some facts on the subject of this complaint, instructive to the practitioner, important to the physiologist, and interesting to the general inquirer into the laws of nature. It states that one person in fifteen, taking the whole mass of society, suffers under this disease: that in the labouring part of the community, the average is one in eight or nine; and that in some particular places of low and damp situation, the proportion may be computed at even a fourth of the labouring population. In upwards of three thousand cases, 741 were double hernia; of these double hernia 47 were *femoral*; and of these 47, three were in males, and forty-four in females; 694 were *inguinal*: of these, 609 occurred in the male, and 85

in the female. Of the single hernia, in number 2272, fifty-seven male femoral, and 163 female femoral occurred; 1520 male inguinal, and 399 female inguinal. Out of the aggregate number there were 133 umbilical; of these 36 appertained to the male, and 97 to the female. Of the single hernia, more than two-thirds happened on the right side. A small proportion of triple, and other extraordinary cases occurred; but they were extremely rare, and mostly existed among the female sex.

The talent for invention, the lively genius of the French, and their extensive intercourse with all nations, combine to give to the progress of their scientific literature an extent and an interest, that scarcely any other people reach or excite. In this abundant field we are ashamed to have gathered so little; but the time alloted to the task, like true patriots, we have almost exhausted on our native soil; and have thus done an injustice to our versatile neighbours, which, however, we hope another year to repair by an ample compensation.

The prominent feature in the medical literature of France, during 1806, as in that of England, has been the progress of VACCINATION. On the 12th of July, the central vaccine committee of Paris made their report on the propagation of vaccine inoculation in that country during the last 12 months. From this report it appears, the number of individuals vaccinated in the 42 departments, during that period, amounts to 125,992. Whether the vaccinated have been exposed to variola by inoculation, or by an intimate commerce with those labouring under the disease, they have equally and uniformly, where they have regularly gone through the vaccine infection, resisted variolous contagion. The most important result of the report of the committee is the certainty of the progressive diminution of mortality wherever vaccination has been introduced, and the increase of deaths where it has been neglected.

The substitution of carpenter's glue for cinchona, in the cure of intermittents, is one of those strange appropriations we must reject with silent neglect, if the evidence of the fact be not the most decisive and satisfactory. Some experiments on cinchona, led Sequin to conclude it contained gelatine; and this gave him the idea of curing intermittents by the use of glue. Dr. Gautieri details a history of the experiments made with this substance in Italy, and states their success; Dr. Bisckoff confirms its powers by a similar detail of facts, occurring in Germany. And from this we are led to understand, and pressed to believe, that the the almost specific qualities of the Peruvian bark, are to be found in simple animal, or, perhaps, vegetable gelatine.

We must look back a year or two, to notice a very ingenious work on the revolutions of medical science, by Cabanis, especially as it has this year appeared in an English dress.* The profound philosophical views, the learning, and the science manifested in this work, sufficiently recommend it to the attention of the public. Englishmen will see with surprise and regret, that this inquirer into the History of Science, has only *just* heard of Harvey and Sydenham; that the treasures of medical literature in the English language are, to him, nearly a blank. This is only to be accounted for, from the want of some work, which would give a general view of the medical learning of this country.

A favorite method of propagating and improving science in France, is by giving prizes for the best dissertations on specified subjects. This has taken place in a smaller degree in England, where it might be extended in a manner that would meliorate science, be the means of bringing genius into action, and become the reward of merit. In Paris, and in the departments, a variety of these prizes are annually announced. For 1806, the Parisian Pharmaceutic Society has offered two prizes for answers to ninepharmaceutic questions.† The editors of the *Gazette de Santé* have given a gold medal for the best answer to the following questions: What are the proximate causes of Epidemics? Do they depend upon various miasmata conveyed through the air, or are they occasioned by actual contact? Do they depend merely on the qualities of smells,

---

* A Sketch of the Revolutions of Medical Science, and Views relating to its Reform. Translated from the French of P. J. G. Cabanis, by A. Henderson, M. D. with notes. 8vo Lond. 1806. pp. 420.

† 1. Does there exist a process for constantly obtaining kermes of the same colour and quantity? 2. What is the reason that whey does not always clarify of the same colour? 3. Which is the best process for obtaining the purest and most energetic emetic? 4. What is the difference between electuaries recently prepared, and those which are several years old? 5. Which is the best method of preserving the various parts of plants, in regard to their *aroma*, colour, &c.? 6. What is the best method of ascertaining the natural families of plants, from their chemical affinities? 7. What is the best method of preparing distilled waters from aromatic plants? 8. Which are the extracts that should be made from green, and which from dried plants? 9. The products of the infusion or decoction of inodorous substances not being the same, in what does that difference consist?

smells, or is it clear that all stimulants are preservatives against infection? The Medical Society of Lyons has announced a prize for the best disputation explaining the diagnostic and prognostic signs afforded by the state of the tongue, lips, and teeth, in acute and chronic disorders; and of the advantages that may result to practice from the discovery. The Medical Society of Brussels, re-established in 1804, has been employed in medical and philosophical researches of great importance. Their prize question for this year is, "What are the characteristic symptoms of inflammation of the mucous system, and what are the phenomena consequent to such inflammation in regard to the organs in which it takes place, and the proper treatment in such cases?"

Professor Dumas, of Montpellier, in an ingenious inquiry into some peculiarities in the structure and functions of animal bodies, has proved, by a number of facts, that the different organs of the human frame have the singular property of being transformed into each other. He distinguishes four species of this transformation. The 1st species comprehends changes in the natural form of the organ. The spleen sometimes assumes the colour and form of the liver, and the duodenum is distended to the size of the stomach. The second species relates to changes in the chemical composition of the organs. The change in the chemical principles of an organ are, either in its albuminous matter, in its fibrous texture, in the gelatine, or in the earthy calcareous salts It is a well known fact, that after death, the muscles sometimes change to a fatty substance resembling spermaceti. M. Dumas has seen the same change occur during life. In dissecting the body of a man who died of catarrhal fever, he found the anterior muscles of the chest, some of those of the face, those of the shoulders and the arms, reduced to this substance; in the muscles of the lower part of the trunk, and of the thighs and legs, this transformation was in the act of taking place. The lungs have been changed into a substance resembling liver; the bones have been transformed into cartilage, and the arteries have become bone. Of the 3d species, the change in organic structure, many instances are related. The 4th species, under which is arranged the transformation of the vital functions, contain many instances of singular deviation from the regular course of nature. Haller mentions a man, who after a nervous affection, acquired such an increase of misplaced sensibility, that all the organs of the body became auditory, distinguishing the properties properties and the force of sounds, with the same correctness as the ear. M. Dumas has seen at Montpellier, a young woman, who during the paroxysm of catalepsy,* had a concentration of sensibility in the præcordia, where the organs of sense seemed for a time to be fixed; she related, that in the paroxysm, the stomach had all the properties of the eye and the ear. The functions of the uterus, in the catamenial evacuation, have often been supplied by very distant organs. The vessels of the nose, the eyes, the umbilicus, the anus, the stomach, the lungs, &c. have, at times, poured out this periodical discharge.

The hymen, a membrane to which such importance has been attached, but the existence of which some anatomists have doubted, has lately been shown by Duvernoy, not to be peculiar to the human female, but to be common to the females of the class Mammalia.

From Spain we have received intelligence of peculiar interest. In the Madrid Gazette of the 14th of October, 1806, there appeared a detail of a voyage round the world, undertaken by the order of the Spanish government, for the purpose of disseminating *Vaccine Inoculation.* Dr. Francis Xavier Balmis, surgeon extraordinary to the King, conducted this humane expedition, and he returned to Old Spain on the 7th of September, 1806, after having carried, or been the means of carrying, the vaccine fluid to the greater part of the continents of Asia, Africa, and America. It had been a point of no small difficulty, and in some voyages of unusual length, it was thought impossible to convey the vaccine fluid in an active state; but in this voyage an effectual expedient was adopted. Twenty-two children, who had never undergone the small-pox, were selected for the purpose of transmitting this fluid in an active state. These children were vaccinated at proper intervals during the voyage, and thus a supply of the infecting material was secured.

Dr.

---

* The extraordinary and almost supernatural appearances that occur in the cataleptic paroxysm, have led, in ages less enlightened than the present, to a belief in the agency of dæmons, and the influence of witchcraft. In some instances, while the patient seemed but breathing marble, the most exalted and elaborate mental action was going on. We have, in the progression of a cataleptic case, seen so many unusual deviations from the common laws of nature, both mental and corporeal, that, were these pages not appropriated to another purpose, we should venture to give a full detail of its history.

Dr. Balmis has brought with him a considerable collection of exotic plants, and drawings of valuable subjects in natural history. He has also amassed much important information, for the naturalist, the physiologist, and the agriculturist

The circumstances that have compelled us to touch but slightly on the progress of the medical art in France, operate with additional force against our going minutely into the extensive field which Germany and the North presents. From this immense store-house of hypothetical and practical knowledge, we cannot refuse, however, to extract some articles of more than common interest. The system of Gall continues to excite sensations, and to divide the continent into two parties; the press teems with Anti-Galls and travels of Craniologists; physicians of eminence are ranged on each side; the celebrated Hufeland defends, and Walter, the anatomist of Berlin, opposes this system of deep science, *or of wild speculation.* Though the discriminating good sense of our countrymen will forbid the system of Gall making converts, or finding advocates in the British isles, yet so singularly whimsical is the hypothesis, and so boldly are its powers asserted, that a short statement of its leading principles will at least amuse. Gall pretends to have arrived, by observations, at a knowledge of the principal faculties of man, his inclinations, and his passions, by a mere inspection of the *Cranium.* He distinguishes upon the cranium different eminences, and each of these eminences, in his system, indicate a leading faculty, a master passion, or a predominant inclination. Dr. Friedlander has given a summary of this doctrine. Gall, in imitation of other great apostles, does not condescend to explain himself. He writes not, but his pupils descant on his principles, and develope his system. The eminences on the cranium, or organs, as they are called in this system, are in number 27. The 1st of these organs indicates the faculty of sexual intercourse; the 2d, is the organ of parental affection; the 3d, of docility, or teachable disposition; the 4th, memory of places; 5th, memory of persons; 6th, knowledge of colours; 7th, talent for music; 8th, for arithmetick; 9th and 10th, for words and language; 11th, the arts of design; 12th, for friendship; 13th, 14th, and 15th, for fighting, murder, and cunning; 16th, for theft; 17th and 18th, pride and vanity; 19th, 20th, and 21st, circumspection, comparison, and judgment; 22d, 23d, and 24th, wit, the power of induction, and good nature; 25th and 26th, are the organs of of theosophy and constancy; and the 27th is the organ of perspicuity, or the faculty of clearly explaining our ideas.*

In the usual course of anatomical studies, (for we consider the equivocal pursuits of Gall as deviating too much from the direct road of science to be arranged with that useful art) dissertations and observations on subjects taken from human and comparative anatomy, by Michel, have appeared; observations anatomico-physiologica, by Okle; commentatio anatomica-physiologica, medico-chirurgica de morbis occulorum, by Paterka; chirurgisen anatomische abbildungen, by Rosenmüller; and Sandifort's Deglutionis Mechanismus Verticalisectione Narium, Oris, Faucium Illustratus, are among the publications of this year. *Beer*, the German Scarpa, has communicated some further observations on staphylomatose diseases of the eye; *Wickelhausen* has written on the symptoms, prevention, and cure of pituitous pulmonary consumption; *Wollkop* has published a second volume on Dysentery; *Vogel's* anthropological and medical observations; *Frank's* work de curandis hominum morbis epitome; *Embden* versuch einer hypochondralogia; and *Lehnkosinük* on the influence of the passions and affections of the mind, with respect to the cause and cure of diseases; with a second edition of *Keil* on the cure and symptoms of fever, are likewise among the publications of this year. *Hufeland* has also announced a fourth volume of his System of Practical Medicine, and the second section of his Therapeutics, treating of cutaneous diseases and on the effects of poison.

Medical jurisprudence is a branch of professional science to which the English have been singularly inattentive, while the practitioners on the continent have explained its uses, both by public lectures and through the medium of of the press. Among the productions on this subject, are Medical Memorabilia for the use of judges, physicians, and clergymen; and a general Repository for medical Police, published periodically by *Scherf*.

An instrument has been invented by Dr. Bozzini, of Frankfort, which promises to be extremely useful to the surgeon. To this instrument he gives the name of "*light spreader*," and its object is to afford an inspection of the interior of wounds, and various parts of the human body, as the œsophagus, vagina, and uterus. A description of it, with drawings, is intended to be published.

The

* Med. and Phys. Journ. vol. xv. p. 201.

The continent of America is interesting to the naturalist and the physician in an extreme degree. The unexplored treasures of its immeasurable wastes, its extensive savanna's, and its immense forests, awaken the curiosity of the botanist and the zoologist; while the new forms of disease, or the magnified state of some morbid affections occasionally seen in the old world, are of equal importance to the inquirer into medical science. The singularity of the external form of the *ab origines* of this vast country, the wild and terrible novelty of their customs, and the powerful remedies which they are said to possess, are circumstances of no trivial concern to the philosophical physician. The region that has given origin to Syphilis, and produces the cinchona, can never sink into mediocrity in the history of science. The Anglo-American has derived from the stock from which he sprung, the talent for the study of science, and the investigation of Nature and her laws. Rush, Barton, and Mitchell, are names that would not discredit the universities of Europe. Through every district of the United States, schools of science are established; among these the medical art evidently takes the lead. "The prevalence of malignant and mortal epidemics, within the last fifteen years, has produced in the population of America, a deeper conviction of the dignity and value of the medical profession: and has also awakened among physicians themselves a more ardent spirit of research and investigation, and has impelled them into paths in which professional reputation and usefulness are alone to be found. It has drawn them into controversies, which, being keenly agitated, and putting all their powers on the stretch, have produced bolder inquiries, more ingenious and more discriminating theories, more precise and logical habits of thinking." The conjunction of this stimulus to exertion, with the rapid progress of the medical school at Philadelphia, and the establishment of three periodical publications* on medicine, will account for the pre-eminence of this branch of natural science.

In

* The Medical Repository, and Review of American Publications on Medicine, Surgery, and the auxiliary Branches of Science, by Drs. Miller and Mitchell, of New York, has compleated its 8th volume.——The Philadelphia Medical Museum, in quarterly numbers, conducted by Dr. Redman Coxe, has arrived at a 2d volume.——The Philadelphia Medical and Physical Journal, collected and arranged by Prof. Barton, of the University of Pensylvania, is also proceeding in a manner that cannot fail to be useful to science.

In addition to the regular periodical publications, we notice in the year 1806, a Pharmacopœia of the United States,* by Dr. Coxe, executed in a manner that places his industry and his judgment in a most reputable light; and a volume of inaugural theses,† containing fourteen dissertations; on the influenza; on the salutary effects of mercury in malignant fevers; on digestion; experiments and observations on the absorption of active medicines into the circulation; on dysentery, by an induction of facts from which the Mitchilian doctrine of pestilential fluids is illustrated; on external applications; on the properties of polygala seneka; on the mutual influence of habits and disease; on the cause of the extensive inflammation which attacks wounded cavities and their contents; on the lupulus communis; on wounds of the intestines; a chemico-physiological essay, disproving the existence of an aeriform function in the skin, and pointing out, by experiment, the impropriety of ascribing absorption to the external surface of the body; on cutaneous absorption; an attempt to prove that lues venerea was not introduced into Europe from America; an experimental inquiry into the modus operandi of mercury, in curing lues; an experimental proof that lues venerea and gonorrhœa are two distinct forms of disease.

In the slightest observations on the progress of Medicine in America, it will be expected that some attention should be given to *typhus icteroides*. The experience of the physicians of the West Indies, and of N. and S. America, point them out as the best qualified to give a practical account of this dreadful scourge: and to them we must look for an authentic detail of its phenomena. There are three circumstances in the history of yellow fever, that are interesting in different degrees:—the extent of its ravages, the progression of its symptoms, and its origin. At present, we shall only notice the state of opinion on the latter. To ascertain whether the yellow fever of America is of domestic growth, or whether it has been imported from another region; whether it is contagious, or disseminated by some other principle, are questions of the utmost weight and importance; and we find that they have undergone the

* American Dispensatory. By Dr. Redman Coxe. 8vo. Phil. 1806. pp. 800.

† Medical Theses, selected from among the inaugural dissertations published and defended by the graduates in medicine of the university of Pensylvania. Edited by C. Caldwell, M. D. 8vo. Phil. 1806. pp. 396.

the most animated and enlightened discussions. These discussions have produced a very singular state of public opinion. Nineteen physicians at least in twenty, in the United States and the West Indies, maintain, it is very positively asserted, the domestic origin and non-contagious nature of this disease; while a great majority of the populace in the commercial towns, and the merchants, believe it contagious, and that it is imported from abroad. If there is one question in medicine of more difficult solution than another, and in which an erroneous opinion is of the most fatal importance, it is the question of the existence of contagion. And yet so boldly incautious is mankind, that we have seen in every age the most strenuous efforts made to prove that even pestis is not contagious; and that the laws of quarantine, and every restrictive ordinance, are both useless and absurd. So seriously important is this question, however, that error on either side, will involve destruction of life or great commercial loss. In this dilemma, it cannot be difficult to see to which side the prudent physician will be biased. It is his business to preserve life; and he will ever consider that if he errs, he errs on the safe side when he admits, in sweeping epidemics like the American yellow fever, the presence of contagion. If this fever is proved by a clear induction of facts, not to be contagious or of foreign origin, the question, with regard to it, is decided: but the use of quarantine, generally, as the means of checking the spread of disease, we shall continue to defend until we see more distinctly the non-existence of contagion, and that every epidemic is of domestic origin.

We cannot close this imperfect view of the progress of medical science in the year 1806, without observing, as an apology for its omissions, and in extenuation of its faults, that it has been composed on the "spur of the occasion;" in moments snatched from serious professional employments; when materials were to be hastily collected, and arranged almost without revision: that it has been written, not in ease and quiet, but under sudden and frequent interruptions; when a happy train of thought, or felicity of language, if it ever occurred, has been dissipated and lost. If it has failed in perspicuity, if it is erroneous in point of fact, or essentially false in opinion, a hope is cherished that its imperfections may be occasioned by the short time allotted to its compilation: and that another year, when leisure will allow a wider range for the collection of materials, a deeper investigation of their properties, and a nicer nicer discrimination of language; a detailmay be produced worthy of the subject, more reputable to the author, and deserving the patronage of a profession, whose good opinion he is solicitous, above all things, to merit.

*Princes Street, Cavendish Square, June,* 1807.

